# Comparison of medicines management strategies in insurance schemes in middle-income countries: four case studies

**DOI:** 10.1186/s40545-017-0105-y

**Published:** 2017-05-16

**Authors:** Warren A. Kaplan, Paul G. Ashigbie, Mohamad I. Brooks, Veronika J. Wirtz

**Affiliations:** 1 Department of Global Health, Boston University School of Public Health, Crosstown Center, Room CT-363, 801 Massachusetts Avenue, Boston, Massachusetts 02118 USA; 2 Pathfinder International, 9 Galen Street, Suite 217, Watertown, 02472 Massachusetts USA

**Keywords:** Health insurance, Drugs, Middle-income countries, China, Indonesia, Ghana, Mexico, Universal health coverage

## Abstract

**Background:**

Many middle-income countries are scaling up health insurance schemes to provide financial protection and access to affordable medicines to poor and uninsured populations. Although there is a wealth of evidence on how high income countries with mature insurance schemes manage cost-effective use of medicines, there is limited evidence on the strategies used in middle-income countries. This paper compares the medicines management strategies that four insurance schemes in middle-income countries use to improve access and cost-effective use of medicines among beneficiaries.

**Methods:**

We compare key strategies promoting cost-effective medicines use in the New Rural Cooperative Medical Scheme (NCMS) in China, National Health Insurance Scheme in Ghana, Jamkesmas in Indonesia and Seguro Popular in Mexico. Through the peer-reviewed and grey literature as of late 2013, we identified strategies that met our inclusion criteria as well as any evidence showing if, and/or how, these strategies affected medicines management. Stakeholders involved and affected by medicines coverage policies in these insurance schemes were asked to provide relevant documents describing the medicines related aspects of these insurance programs. We also asked them specifically to identify publications discussing the unintended consequences of the strategies implemented.

**Results:**

Use of formularies, bulk procurement, standard treatment guidelines and separation of prescribing and dispensing were present in all four schemes. Also, increased transparency through publication of tender agreements and procurement prices was introduced in all four. Common strategies shared by three out of four schemes were medicine price negotiation or rebates, generic reference pricing, fixed salaries for prescribers, accredited preferred provider network, disease management programs, and monitoring of medicines purchases. Cost-sharing and payment for performance was rarely used. There was a lack of performance monitoring strategies in all schemes.

**Conclusions:**

Most of the strategies used in the insurance schemes focus on containing expenditure growth, including budget caps on pharmaceutical expenditures (Mexico) and ceiling prices on medicines (all four countries). There were few strategies targeting quality improvement as healthcare providers are mostly paid through fixed salaries, irrespective of the quality of their prescribing or the health outcomes actually achieved. Monitoring healthcare system performance has received little attention.

## Background

Over the last decade international agencies and individual countries have shown commitment to promote universal health coverage (UHC), defined as: “[…] ensuring that all people have access to needed promotive, preventive, curative and rehabilitative health services, of sufficient quality to be effective, while also ensuring that people do not suffer financial hardship when paying for these services” [1].

The World Health Organization (WHO) defines a series of necessary conditions to achieve UHC, one of which relates to medicines - “Access to essential medicines and technologies to diagnose and treat medical problems.” Various authors have described in more detail how to define access and how to measure it [2, 3]. Nonetheless, how to balance medicines access, affordability, quality and sustainability of supply, has played a relatively minor role in discussions regarding UHC and the necessary conditions needed to achieve it [4]. Medicines management is critical to successful implementation of UHC but, until recently, few studies exist guiding policy development and implementation in low and middle-income countries.

Accordingly, we employ a case-based approach to study what strategies payers use to promote cost-effective use of medicines. We have chosen four insurance schemes in Mexico, China, Ghana and Indonesia, which provide coverage for poor and/or underserved populations. We compare the medicines management strategies to promote cost-effective use of medicines in these insurance schemes and discuss challenges in their implementation.

## Methods

### Selection of the countries

We chose four countries (China, Indonesia, Ghana and Mexico) which (1) are at different stages of development with regard to UHC of their population; (2) have different funding arrangements (e.g. social health insurance (SHI) or tax-based systems), and (3) are from different geographical regions. In addition, we also considered country income level and whether the health service providers are public or private.

### Literature review and stakeholder interviews

We carried out a desk review of relevant peer-reviewed and grey literature related to UHC and medicines published between 2000 and 2013 for each of the four countries. To complement our review we conducted interviews with stakeholders (3 in China; 4 in Ghana, 1 each in Indonesia and Mexico) involved in developing medicines coverage policies or affected by these policies. We sought the perception of these stakeholders regarding relevant publications as well as administrative documents describing the medicines- related aspects of the health benefit or insurance programs. We also asked them specifically to identify publications discussing the unintended consequences of medicines coverage and pharmaceutical management policies in place.

### Conceptual framework for analysis

Analytically, the medicines and finance policies used to balance access, affordability, quality and sustainability were divided into the following five broad categories [5]: (1) selection, (2) procurement, (3) contracting, (4) utilization management, and (5) monitoring member satisfaction, and purchasing and prescribing patterns.

For each of these broad categories, the public and private sectors have often-times competing interests with regard to UHC [4]. These interests are: (1) keeping costs affordable, (2) ensuring availability of quality generic and innovator products, (3) improving equitable access, and (4) ensuring appropriate use.

## Results

### Brief summary of scaling up health insurance coverage in each country studied

Table 1 describes the countries included and their respective characteristics as of 2014. They represent a range of insurance coverage from 39% in Ghana to approximately 100% for Mexico and China. Two countries have UHC with only government revenue funding (Indonesia, Mexico), two a mix of government revenues and beneficiary contribution (Ghana, China). Each country is from a different WHO Region. Two countries are lower-middle (Indonesia, Ghana) and two are upper-middle income countries (Mexico, China). An overview of the demographic, health and health care related indicators of the four countries chosen can be found in Appendix.

**Table 1:** Countries selected as case studies and the characteristics as of 2014

Country	Development stage of UHC^a^ % population covered in the entire country^a^	Funding arrangement (year of inception)^b^	Geographical region (WHO region)	Country income level	Provider mix^b^
China	90%	NRCMS (2003): Premiums and federal and local government subsidies URBMI (2007): Premiums and government subsidies	WPR	UMIC	NRCMS: Largely private contractors URBMI: Largely public
Indonesia	40–63%	JAMKESMAS (2004): Government revenues	SEAR	LMIC	Jamkesmas: Nearly exclusively public
Ghana	39%	NHIS (2004): Social Health Insurance	AFR	LMIC	NHIS: Mixed public/private providers
Mexico	80–100%	SP (2003): Premiums and taxes	AMR	UMIC	SP: Nearly exclusively public

^a^The percentage of the population covered varies by data source and method of estimating coverage; hence, we report for some countries a range. Percentage coverage is based on most recent reporting or 2014, whichever is later

^b^This information refers to the reform program that specifically targets the poor population: *NRCMS* New Rural Coorperative Medical Scheme, *URBMI* Urban Residence Basic Medical Insurance, *MoHME* Ministry of Health and Medical Education, *NHIS* National Health Insurance Scheme, *RHI* Rural Health Insurance, *SP* Seguro Popular

Region: *WPR* Western Pacific Region, *SEAR* South-east Asian Region, *AFR* African Region, *AMR* Region of the Americas, *EMR* Eastern Mediterranean Region

#### China

In China, three major health insurance programs cover specific groups: rural residents under the New Rural Cooperative Medical Scheme (NCMS), urban employees under the Urban Employees Basic Medical Insurance (UE-BMI), and unemployed urban residents under the Urban Residents Basic Medical Insurance (UR-BMI). We note that in early 2016, China announced the decision to merge the UR-BMI and NCMS schemes [6]. There will be unified coverage, a fund pooling mechanism, a benefits package and reimbursement rates, a basic medical insurance drug list, unified selection of health providers, and fund management. For the purpose of this retrospective study, only the NCMS will be analyzed as no evaluation has been made of the combined UR-BMI and NCMS schemes. Various experiments are also taking place at lower levels, giving the entire Chinese insurance system a very dynamic nature. The NCMS incorporates voluntary enrollment and coverage of catastrophic illnesses. Apart from these two requirements, the design and implementation of the program is left to local governments. An extensive survey in Western and Central China found the most common model of NCMS combines a medical savings account (MSA) and high-deductible catastrophic insurance for inpatient services. Eighty percent of the 10 RMB premium (about 1.5 USD as of this writing), is put into an MSA to pay for outpatient visits and can be shared among household members. The government’s 20 RMB subsidy plus the remaining 2 RMB premium are pooled to cover inpatient hospital expenses above a certain deductible. The amount of the deductible varies by locale, with the majority of them above 400 RMB. Besides the deductible, patients still have to pay 40–60% of covered inpatient expenses. The benefit package also caps the benefit payment at 10,000–20,000 RMB. NCMS risk-pooling is at the county level, not the village level.

#### Indonesia

In 2005, the Askeskin program provided basic health coverage and medicines to the poor. This health insurance program was later expanded to include the near-poor in 2007 and renamed as Jamkesmas. The Jamkesmas program in 2012 had over 76 million beneficiaries – a third of the national population – and was the largest health insurance scheme in Indonesia [7]. Two other social health insurance schemes also existed in Indonesia: Askes was targeted to civil servants and had 17 million beneficiaries while Jamsostek enrolled 5 million employees in the private sector [7]. Combined, the three social health insurance programs covered 40% of the Indonesian population in 2012. However, the analysis in this study only looked at the Jamkesmas program from 2012–2013 and its medicines related benefits. We note that the Indonesian government enacted the Badan Penylenggara Jaminan Sosial (BPJS) law (Law No. 24/2011) in 2011 which was intended to unify all social health insurance programs under one not-for-profit administrator in 2014. The Indonesian government rolled out the Jaminan Kesehatan National (JKN) program on January 1, 2014, with the ambition to achieve national UHC by January 2019 [8].

#### Ghana

In 2003, the National Health Insurance Authority (NHIA) was established as the regulator and the implementer of all health insurance schemes in the country (National Health Insurance Scheme (NHIS)). The NHIS is primarily financed by a 2.5 percentage top up of the Value Added Tax (National Health Insurance Levy) and a 2.5% levy of social security contributions made by formal sector employees (involuntary payroll deductions), and premiums paid by informal sector workers [9]. Both public and private health facilities are accredited to provide services under coverage by the NHIS. More than 50% of all patients, whether using the NHIS or not, seek care from the private sector [10]. These private institutions include for-profit standalone pharmacies and licensed chemical sellers, for profit hospitals and clinics and not-for-profit health providers (mission hospitals). Mission health facilities which account for a substantial proportion of district hospitals in the country are usually described as private not for profit but their health workers are on government employee payroll and they also benefit from government programs meant for the public sector. In total, government support to overall expenditure of mission health facilities budget is 34–35%; the remaining is internally generated. In terms of service delivery mission institutions provide 30% of inpatient care and 20% of outpatient care (personal communication from key informant). Apart from the NHIS there are a small number of private insurers which also offer insurance packages with medicines benefits to well-to-do clients and are outside the scope of this study which will focus on NHIS.

#### Mexico

In 2000 around 50% of the population did not have health insurance, mostly those working in the informal sector or the self-employed [11]. In the past, the entitlement of health insurance had been defined by employment status with those in formal employment and their dependents covered by social security [12]. With the creation of the national health insurance program called *Seguro Popular* in 2003 the Mexican government initiated to scale up UHC with the aim to reach 100% population coverage by 2010 (which was later extended to 2011). Affiliation was targeted towards the population previously not covered by social insurance. Official government sources declared 100% coverage in 2012 [13]. Seguro Popular seeks to provide health service coverage, through voluntary, public insurance for persons that are not affiliated to any social security institution. In 2014 it provided coverage for 275 medical interventions, described in the Universal Health Service Catalogue. *Seguro Popular* is operated at national level by the National Commission of Social Protection in Health (CNPSS). At State level the State *Seguro Popular* Fund Holders (REPSS) are responsible to manage funds and purchase care. As the health system is decentralized, the national policies are implemented in a heterogeneous manner throughout the states [14].

### Strategies to promote cost-effective medicines use

For the four programs studied –NCMS, NHIS, Jamkesmas and Seguro Popular- strategies to select medicines were well documented in the literature. In contrast, purchasing, contracting and utilization were less well documented. Table 2 presents a comparison between the four programs.

**Table 2:** Overview of strategies used to promote cost-effective use of medicines in the four medicines benefit programs

Type of strategies	Strategies Medicines	NCMS (China)	National Health Insurance (Ghana)	Jamkesmas (Indonesia)	Seguro Popular (Mexico)
*Selection*	*Formulary*	*✓*	*✓*	*✓*	*✓*
	Cost sharing for medicines included in the formulary	✓	✗	✗	✗
	Generic substitution	✗	✓	✗	✗
*Procurement*	Medicines prices negotiation or rebates	✓	✗^c^	✓	(✓)
	*Bulk procurement*	*✓*	*✓* ^a^	*✓*	*✓*
	Generic reference pricing	✗	✓	✗^a^	✓
*Contracting*	Fee for service for prescribers	✓			
	Fixed salary for prescribers^a^		✓	✓^a^	✓
	Fixed reimbursement rates for medicines	✓	✓	✗	✗
	Preferred provider network (accreditation)	✗	✓	✓	✓
*Utilization*	*Standard treatment guidelines*	(*✓*)	*✓*	*✓*	*✓*
	Payment for performance	(✓)	✗	✗	✗
	*Separation of prescribing and dispensing*	*✓*	*✓*	*✓*	*✓*
	Disease management programs	✓	No info	✓	✓
*Monitoring and evaluation*	User satisfaction monitoring	✗	✗	✗	✓
	Medicines purchasing monitoring	✓^b^		✓	✓
	Prescription monitoring	✗	✗	✗	✗

^a^ = in the public sector; () = Limited use; ^b^ = information not publically available; ^c^ = healthcare facilities do their own negotiation with suppliers; italics = strategies common to all the four insurance programs

The following strategies to promote cost-effective medicines use were common to all the four insurance programs (in bold in Table 2):

- use of formularies (at various levels in the healthcare system),
- bulk procurement,
- use of standard treatment guidelines and
- separation of prescribing and dispensing.

Formularies in the four cases were based on national essential medicines lists but each program adapting or modifying it for its specific needs. For instance, for the NCMS formularies, part of the medicines are selected from the national EML which in 2012 contained 520 medicines, 317 Western and 203 Traditional. Since each province has their own EML (the National EML plus a provincial supplementary list), some medicines are taken from the provincial supplementary list. Programs made a distinction between medicines used at lower levels of care (e.g. primary care) versus those that should be available at high levels of care (second or tertiary care).

For all four programs the bulk procurement was done at regional level or national level (e.g. state or province). Systems to increase transparency of public procurement prices, volumes and bidding process were recently introduced in all of them [15–17]. Standard treatment guidelines had been developed in all four of them [18–21]. However, the extent to which they are implemented into clinical practice and linked to the selection of medicines for inclusion in the formulary varies. For instance, little information was found on the selection criteria of medicines included into the provincial formularies of NCMS and the establishment of evidence-based guidelines in contrast to experience-based care is a challenge in many settings [18]. In contrast, the formulary of Seguro Popular refers to clinical guidelines [22].

Furthermore, medicine price negotiation or rebates, generic reference pricing, fixed salaries for prescribers, accredited preferred provider network, disease management programs and monitoring of medicines purchases were implemented by three of the four schemes.

In Ghana, payment structures for public sector prescribers and dispensers is based on a policy which places all public service employees on a single vertical salary structure [23]. Thus prescribers are paid fixed salaries, irrespective of the quality and volume of services rendered. In Indonesia public health workers, including government physicians and pharmacists, receive a fixed salary independent of productivity or capitation [24]. In Mexico, prescribers in the public sector that provide services for Seguro Popular beneficiaries are paid by fixed salaries and do not receive any payment related to services provided (no financial incentives nor disincentives). Many physicians working in public health units also have their private consultancy offices [25].

In China, payment was linked to volume and type of dispensing services [15, 26, 27]. Whereas Indonesia [7] and Mexico (information provided by key informant) had fixed salaries for those filling prescriptions in the public sector, dispensing charges in China were included in the product reimbursements [27, 28]. There were no dispensing fees in Ghana [29].

There was a lack of information on payment for performance, monitoring user satisfaction and prescription monitoring.

Jamkesmas and Seguro Popular share many common strategies. In the case of Jamkesmas in Indonesia [7] and Seguro Popular in Mexico most providers (prescribers and dispensers) are located in public clinics that operate under the provincial or state directed Ministry of Health [30]. Dispensing outlets and pharmacies are within the clinics. The decentralization of the health system in Indonesia and in Mexico results in variations in the pharmaceutical policy strategies implemented by provincial Jamkesmas administration [31] and state Seguro Popular Fundholders [32]. One important way in which Jamkesmas differs is in the variation in medicines procurement prices among provinces [33]. In Seguro Popular states are mandated to procure at a price that does not exceed a certain amount [34]. In addition to public and private hospitals and clinics, the NHIS in Ghana contracts private standalone pharmacies, and licensed chemical sellers to dispense medicines [29].

The rapid changes and large regional differences in the NCMS in China have made it difficult to describe the current general strategies to promote access and utilization via this insurance scheme. Users have to pay a deductible before the schemes start covering medicines [28, 35]. The coverage has a cap after a maximum insurance payment has been reached in a given time frame [28, 35, 36].

#### Reported impact of strategies of promote cost-effective use of medicines

With regard to China, in terms of the consequences of financing strategies on availability, access, and use of medicines, as well as on household and health system affordability, early cross-sectional appraisals of the health reforms found lower medicines prices in primary care facilities [37]. However, impacts of the health reform on generally low availability [38], total number of prescriptions [39, 40] or less-than-appropriate use [41] was not clear.

In Ghana, expenditures on medicines had increased after the introduction of NHIS [42, 43]. Supplier induced demand for medicines in private hospitals have also been documented [44]. Another study has shown that enrollment patterns did not match well with the medicines utilization changes and the authors have questioned whether the increased spending has improved equity in access and appropriate use [45].

In Ghana and Indonesia, key informants considered the claims-processing systems inefficient, which is often paper-based rather than electronic, and require resource-consuming reviews. Inefficient claims-review systems can lead to delays in payments to facilities, shortages of facility funds to purchase medicines, and medicines stock-outs [9].

In Mexico, no decrease in household expenditure on medicines was found 10 months after the introduction of the insurance scheme [46] or no statistically significant difference in household expenditure was found in comparison to households not insured by Seguro Popular [47].

## Discussion

This study compares strategies to promote cost-effective use of medicines in insurance schemes targeting poor populations in middle-income countries. Whereas medicines policies to manage medicines in insurance schemes in high-income countries are well documented there is a lack of evidence from low- and middle-income countries [48, 49]. Transferability of evidence from high-income settings is limited and the creation of knowledge from low- and middle-income countries is relevant particularly given that many of them are making strides to move towards UHC. Our study contributes to the creation of evidence by analyzing four schemes operating in middle-income countries as case studies.

The results show that the identified country strategies aim to contain expenditure growth through budget caps on pharmaceutical expenditures (Mexico) and medicines price limits (all four countries). Moreover, all four schemes use strategies for cost containment through selection, bulk procurement and standard treatment guidelines. Price negotiation and rebates are also very common (China, Indonesia and Mexico). All these policies and practices including tendering are also commonly implemented in high-income settings [49].

However, the results show that there are a series of challenges in policies that aim to improve performance e.g. providers are mostly paid through fixed salaries, irrespective of the quality of their prescribing efficiency or the health outcomes actually achieved. On one hand, fixed salaries can protect against financial incentives to over-prescribe certain medicines for which prescribers receive a bonus [50]. On the other side, lack of incentives reinforcing good performance or sanctions to impede low quality can result in inadequate prescribing [51]. Policies to improve quality improvement are often more challenging to implement effectively; for instance, payment for performance requires advanced information systems to collect data on provider performance; the availability of such systems is usually limited in middle-income countries [52].

Three of the four schemes do not have cost sharing systems in place, in other words, beneficiaries receive the medicines included in the benefit package free of any co-payment. This protected individuals from financial hardship and out-of-pocket expenditure. An exception was China where there are many different kinds of cost-sharing schemes [53]. It is important to note that there is some evidence of cost-sharing in the Chinese NCMS reducing financial risk of the beneficiaries.

Of the four countries, only Ghana has generic substitution, an advantage in containing costs. Whether it is useful to introduce a generic substitution policy for cost containment depends very much on the context, such as architecture of the pharmaceutical supply and reimbursement system in place. Generic substitution policies are uncommon in systems where the same institution procures, distributes, and dispenses medicines. By design, these schemes procure generic medicines whenever available; originator or brand medicines are not available at the point of dispensing.

Finally, there was little information on any systematic monitoring and publishing performance metrics of medicines prescribing and expenditure by these insurance schemes. For instance, it was unclear how performance of prescribers was monitored, used for feedback or to inform future interventions to promote more appropriate use of medicines. The absence of information is not sufficient to say that schemes do not carry out these activities. In case these insurance schemes lack monitoring systems and performance metrics it will be challenging to implement the aforementioned policies efficiently, track their impact and adjust them if necessary.

The interpretation of the study results should take into consideration the following limitations: the analysis of strategies did not include an assessment of how well they were implemented. In addition, there is limited evidence to evaluate their impact on cost-effective use and access. Furthermore, rapid changes in strategies implemented by each of these insurance schemes make it difficult to keep track and accurately report the status quo. However, this is a limitation that applies to other policy analyses due to the nature of systems that are constantly changing. For certain types of strategies, it was easier to obtain information such as a selection of medicines (e.g. for instance, formularies were publiclly available). In contrast, strategies on procurement and reimbursement of providers were much harder to identify and report on. Hence, underreporting on these strategies is possible. However, we asked stakeholders specifically to provide us with information on documentation gaps to avoid publication bias.

## Conclusions

Moving towards UHC requires countries to promote efficient use of financial resources in all areas of health services including medicines. Insurance schemes in middle-income countries have used a variety of strategies to ensure cost-effective use of medicines; there is no single strategy that will be suitable for all middle-income countries. There is an opportunity for insurance schemes to expand the type of strategies from cost-containment strategies towards those that incentivize quality use of medicines. To that end, we have identified a number of policy gaps that insurance schemes should address, in particular performance based payments and monitoring and performance metrics. Insurance schemes should pay closer attention to these policies.

## Appendix

**Table 3:** Country profiles of four countries included in the case studies (2010)

	Indonesia	China	Ghana	Mexico
Demographics				
Number of inhabitants in 1,000 s	242,326	1,355,243	24,966	114.8
Life expectancy in years 2011				
Male	68	74	62	72
Female	71	77	65	78
Population distribution				
Median age	28	35	21	27
% population under 15	27	19	38	29
% population over 60	8	13	6	9
Economics and health systems financing				
Income group	Lower middle	Upper middle	Lower middle	Upper middle
% total health expenditure of GDP	2.8	5.0	5.2	6.3
% public expenditure of THE	36.1	54.3	58.2	49.0
% pharmaceutical expenditure of THE	1/3			
per capita total expenditure on health (PPP int. $)	123	373	85	962
Health indicators				
Age-standardized mortality rates by NCD (per 100,000 population) 2008	647	604	711	493
Prevalence of raised fasting blood glucose among adults aged ≥ 25 years] (%) 2008				
Male	6.6	9.6	9.9	13.2
Female	7.1	9.4	10.3	14.9
Prevalence of raised blood pressure among adults aged ≥ 25 years 2008				
Male	32.5	29.8	32.7	27.4
Female	29.3	25.6	31.6	21.5
Adults aged ≥20 years who are obese, 2008				
Male	2.5	4.6	4.4	26.7
Female	6.9	6.5	11.7	38.4
Prevalence of smoking any tobacco product among adults aged ≥15 years (%), 2009				
Male	61	51	11	24
Female	5	2	3	8
Alcohol consumption Among adults aged ≥15 years (litres of pure alcohol per person per year) 2008	0.6	5.6	3.1	8.6
Maternal mortality, 2011 Deaths per 100 000 live births	220	37	350	50
Under five mortality, 2011 Total number of such deaths per 1000 live births	151	15	78	16
Vaccination measles Immunization coverage among 1-year-olds (%) 2011	89	99	91	98
HIV prevalence HIV infections per 100 000 population per year	155	…	907	156
Health systems capacity				
Number of physicians per 10,000 inhabitants	2.0	14.6	0.9	19.6
Hospital beds (per 10,000 population)	6	39	9	17
Formal population coverage (% covered by insurance or tax-based arrangements)	40–60%			75–100%
Year of implementation UHC	2005			2003

## Abbreviations

BPJS: Badan penyelenggara jaminan sosial
CNPSS: National commission of social protection in health
JKN: Jaminan kesehatan national
MSA: Medical savings account
NCMS: New rural cooperative medical scheme
NHIA: National health insurance authority
REPSS: State *Seguro Popular* fund holders
UE-BMI: Urban employees basic medical insurance
UHC: Universal health coverage
UR-BMI: Urban residents basic medical insurance
WHO: World Health Organization
